# Identification of Circulating Endocan-1 and Ether Phospholipids as Biomarkers for Complications in Thalassemia Patients

**DOI:** 10.3390/metabo11020070

**Published:** 2021-01-26

**Authors:** Amy Botta, Anik Forest, Caroline Daneault, Kostas Pantopoulos, Adisak Tantiworawit, Arintaya Phrommintikul, Siriporn Chattipakorn, Nipon Chattipakorn, Christine Des Rosiers, Gary Sweeney

**Affiliations:** 1 Department of Biology, York University, Toronto, ON M3J1P3, Canada; amymbotta@gmail.com; 2 Montreal Heart Institute Research Center, Department of Nutrition, Université de Montréal, Montreal, QC H3T 1A8, Canada; anik.forest@mhi-rc.org (A.F.); Caroline.Daneault@icm-mhi.org (C.D.); christine.des.rosiers@mhi-rc.org (C.D.R.); 3 Lady Davis Institute for Medical Research, Jewish General Hospital, Department of Medicine, McGill University, Montreal, QC H3T 1E2, Canada; kostas.pantopoulos@mcgill.ca; 4 Department of Internal Medicine, Faculty of Medicine, Chiang Mai University, Chiang Mai 50200, Thailand; atantiwo@yahoo.com (A.T.); arintayap@yahoo.com (A.P.); 5 Cardiac Electrophysiology Research and Training Center, Faculty of Medicine, Chiang Mai University, Chiang Mai 50200, Thailand; scchattipakorn@gmail.com; 6 Center of Excellence in Cardiac Electrophysiology Research, Department of Physiology, Faculty of Medicine, Chiang Mai University, Chiang Mai 50200, Thailand

**Keywords:** lipidomic, biomarker, diagnostic, therapeutic, thalassemia

## Abstract

Despite advances in our knowledge and attempts to improve therapies, β-thalassemia remains a prevalent disorder with increased risk for the development of cardiomyopathy. Using an untargeted discovery-based lipidomic workflow, we uncovered that transfusion-dependent thalassemia (TDT) patients had a unique circulating lipidomic signature consisting of 387 lipid features, allowing their significant discrimination from healthy controls (Q-value < 0.01). In particular, TDT patients had elevated triacylglycerols and long-chain acylcarnitines, albeit lower ether phospholipids or plasmalogens, sphingomyelins, and cholesterol esters, reminiscent of that previously characterized in cardiometabolic diseases resulting from mitochondrial and peroxisomal dysfunction. Discriminating lipid (sub)classes correlated differentially with clinical parameters, reflecting blood (ether phospholipids) and iron (cholesterol ester) status or heart function (triacylglycerols). We also tested 15 potential serum biomarkers related to cardiometabolic disease and found that both lipocalin-2 and, for the first time, endocan-1 levels were significantly elevated in TDT patients and showed a strong correlation with blood parameters and three ether diacylglycerophosphatidylcholine species. In conclusion, this study identifies new characteristics of TDT patients which may have relevance in developing biomarkers and therapeutics.

## 1. Introduction

β-thalassemia is a group of hereditary disorders that is caused by mutations in globin genes [1]. Annually, around 300,000 children worldwide are born with some form of hemoglobinopathy [2], around 70,000 of them with thalassemia [3]. The disease is characterized by ineffective erythropoiesis due to increased proliferation and impaired differentiation of developing erythroid cells. This triggers expansion of the bone marrow, which can cause bone deformities [4]. In the most severe forms of thalassemia, known as transfusion-dependent thalassemia (TDT) [5,6], patients can only survive with frequent blood transfusions, which lead to secondary iron overload (transfusional siderosis). Moreover, ineffective erythropoiesis promotes increased dietary iron absorption due to suppression of the iron regulatory hormone hepcidin. Thus, TDT patients eventually develop relevant complications such as cardiomyopathy, diabetes, and liver disease, which are managed by iron chelation therapy [7]. Since the early 2000s, the survival rate of patients with thalassemia at age 35 years has been 50% [8]. Novel treatment options, including gene therapy, are emerging [9].

The leading cause of death in TDT is iron overload cardiomyopathy [10], in particular dilated type [11], and patients have varying levels of response to chelation therapy [12]. Previous research has shown that β-thalassemia patients have increased levels of lipocalin 2 (Lcn2), a circulating protein that plays a role in the innate immune system by binding iron and preventing bacterial growth [13]. However, aside from markers for iron overload such as ferritin, liver iron, and cardiac iron, as measured by cardiovascular magnetic resonance T2* (CMR T2*), serum biomarkers are currently not widely available as a diagnostic tool for monitoring the development and progression of cardiomyopathy in thalassemia patients [14].

Before the onset of clinically detectable symptoms, alterations in serum metabolites offers a potential diagnostic biomarker for disease status and predicting response to treatment [15,16,17]. While previous studies identified lipid metabolism as being impacted by the disease and its treatment, the coverage of lipids was limited, mostly to fatty acids. With respect to heart disease and cardiomyopathy, sex-specific differences in both clinical presentation and disease progression have been reported [18,19]. However, very few studies have been conducted to explore the sex-specific differences amongst TDT patients. One of them investigated the differences in myocardial iron and cardiac dysfunction amongst males and females with TDT, and found using cardiac T2 * imaging that there was no obvious sex-specific difference in cardiac iron level or overt differences in cardiac dysfunction [20]. However, a review of previously published literature found that while cardiac levels of iron were similar amongst male and females, female thalassemia patients had decreased cardiac morbidity compared to male patients [21]. It was also found that bone disease is more prevalent amongst male thalassemia patients [22].

Hence, in this study, we examined the impact of TDT and sex on the circulating lipidome utilizing a comprehensive and previously validated untargeted discovery-based lipidomic workflow using high-resolution liquid chromatography-mass spectrometry (LC-MS) [23]. This workflow was applied to a cohort of 61 patients with TDT and 10 healthy controls to assess the impact of disease and sex on the circulating lipidome. We also analyzed whether 15 serum biomarkers of cardiometabolic disease or established clinical parameters correlated with changes in specific lipids. This study design provided new knowledge on potential disease biomarkers as well as new insight on disease pathogenesis.

## 2. Results

### 2.1. Clinical Characteristics of Female vs. Male TDT Patients

Plasma samples were taken from female (n = 33) and male (n = 28) TDT patients, for which the clinical data reflecting blood parameters, iron status, and heart function are shown in Table 1. Both male and female patients had a similar mean age (males: 27 and females: 27) and BMI (males: 19, females: 20), however males were significantly taller than females (*p* = 0.0012). With respect to blood parameters, while there was no significant difference in total white blood cell levels, there were significant differences in composition. Females had higher levels of neutrophils (females: 53%, males: 46%), and lower levels of eosinophils (females: 1.5%, males: 3.4%), basophils (females: 0.6%, males: 1.7%), and monocytes (females: 5.6%, males: 7.6%) compared to males. Both male and female patients had similar levels of serum ferritin (1499 and 1375 ng/dL, respectively), non-transferrin bound iron (NTBI; 6.5 and 7.2 µM, respectively), and CMR T2 * (37 and 40 ms, respectively). In terms of heart function, both male and female patients had similar values, with the exception of left ventricular (LV) diastolic volume, left atrial (LA) size, and systolic (S) from tissue doppler. Males had a higher LV diastolic volume (102 and 79 mL, respectively), larger LA size (3.8 and 3.4 mm, respectively), and higher S value (9.2 and 8.9, respectively). In summary, male and female TDT patients show marginal differences for commonly assessed clinical parameters.

### 2.2. TDT Patients Show Major Circulating Lipidomic Profile Alterations Compared to Healthy Controls

**Figure 1:**
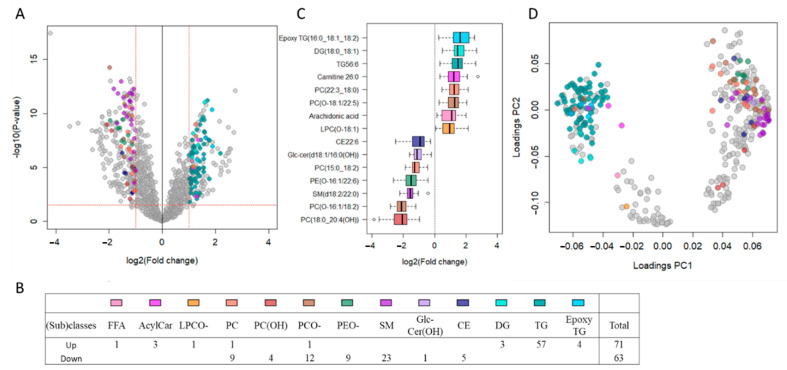
Untargeted lipidomics showed major plasma lipid dyshomeostasis, including increased triacylglycerols and decreased ether phospholipids and sphingomyelins in female thalassemia patients. (**A**) Volcano plot from LC-QTOF-based untargeted lipidomics of plasma from female thalassemia patients (n = 33) compared to healthy female controls (n = 9) depicting the 1463 features obtained following MS data processing. The x axis represents the fold changes (FC; log2) of MS signal intensity values in patients vs. controls, with the y axis representing *p*-values (-log10). Using the selected threshold of *p* < 0.03 (corresponding to Q-value < 0.01; horizontal red line) and FC > 2 or FC < 0.5 (vertical red lines), 387 features significantly discriminated between female TDT patients and controls, of which 136 unique lipids were annotated using our in-house reference database with additional tandem MS, as shown by the color symbols. (**B**) Table listing the 136 annotated lipids that were found to be up- or down-regulated in patients according to lipid (sub)classes and color symbols. (**C**) Box plots of most significant up- or down-regulated annotated lipids for each lipid (sub)class. (**D**) Principal component analysis (PCA) loading plot of the 387 features discriminating thalassemia patients from controls with lipid subclasses for the 136 annotated unique lipids using the color code. Abbreviations: FFA: Free fatty acid; AcylCar: Acylcarnitine; LPCO-: Ether Lysoglycerophosphocholine; PC: Diacylglycerophosphocholine; PC(OH): Oxidized diacylglycerophosphocholine; PCO-: ether diacylglycerophosphocholine, PEO-: ether glycerophosphoethanolamine; SM: Sphingomyelin; Glc-Cer(OH): Oxidized glucosyl sphingolipid; CE: Cholesteryl ester; DG: Diacylglycerol; TG: Triacylglycerol.

We first performed an untargeted lipidomic analysis in female TDT patients (n = 33) and female healthy controls (n = 9; similarly aged; Table 1) to test for the impact of thalassemia on the circulating lipidome, and subsequently tested for sex differences between female and male (n = 28) with TDT. We used a validated untargeted lipidomic workflow with high-resolution liquid chromatography-quadrupole time of flight (LC-QTOF), which allows for the robust analysis of more than 1000 reproducible mass spectrometry (MS) features (defined by a mass-to-charge ratio (m/z), retention time, and signal intensity) in human plasma samples and covers 16 different lipid subclasses in human plasma [23]. Figure 1a depicts the 1463 lipid features obtained following MS data processing using a Volcano plot. Using a subjective stringent threshold of *p*-value < 0.03 (corresponding to a Q-value < 0.01 or false discovery rate (FDR) < 1%) and a fold change (FC) > 2 or <0.5, a total of 387 features discriminated female TDT subjects from healthy controls, of which 228 were lower and 159 higher in TDT subjects (listed in Supplementary Excel File). A total of 136 lipids were annotated using our in-house reference database and additional tandem MS analysis, which corresponds to various lipid (sub)classes identified by the different colored symbols in Figure 1a and detailed in Figure 1b. Figure 1c shows box plots for the most significant up- or down-regulated annotated lipids of each (sub)class, while Figure 1d, which is the principal component analysis (PCA) loading plot for all 387 significant features, including annotated unique lipids shown using the same color symbols, illustrates the similar pattern of changes for lipids within a given lipid (sub)class. We found no significant differences in any lipid features between male versus female TDT patients, at least not based on the selected threshold. Supplementary Figure S1 depicts the 1463 features obtained following data processing of the dataset, of which 59 features passed a threshold of *p*-value < 0.05 and FC > 1.25 and FC > 0.8, albeit all these features had Q-values > 0.50 (corresponding to FDR > 50%) (See Supplementary Excel File 1&2 for the list of 59 features). Among lipids having *p*-values < 0.05 include diacylglycerophosphocholine (PCs), ether PCs, and diacylglycerophosphoethanolamine (PEs) bearing n3 and n6 polyunsaturated fatty acids in sn2 position, which were approximately 30% higher or lower in male than female TDT respectively, concurring with our previous findings in a cohort of healthy individuals using the same lipidomic workflow [23].

**Figure 2:**
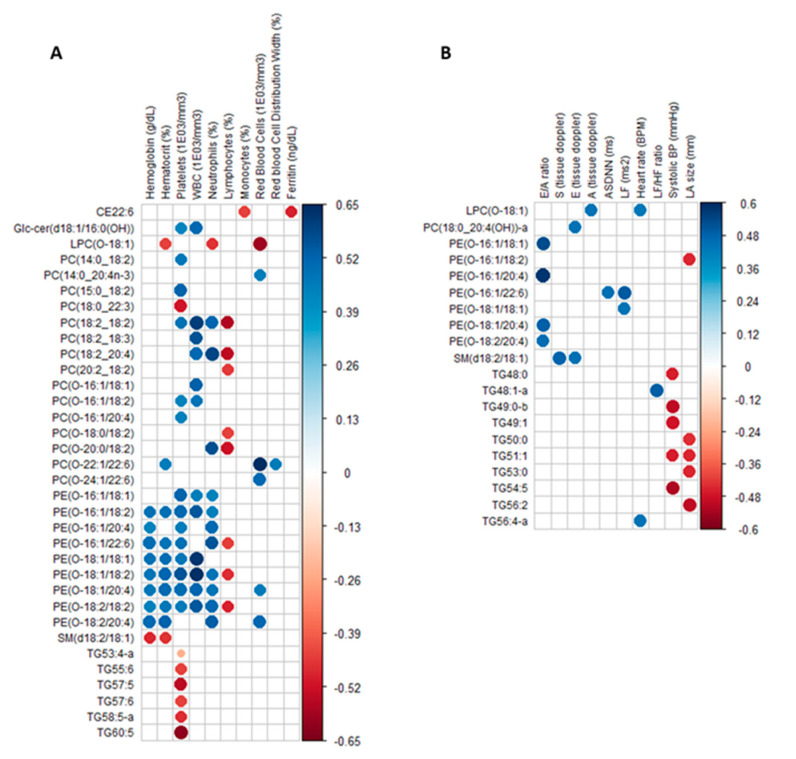
Circulating lipids discriminating female thalassemia patients from controls are differentially correlated to clinical parameters. A correlation matrix was built using the 136 annotated plasma lipids that discriminated female thalassemia patients from controls with a Q-value < 0.01 and FC > 2 or <0.5, as well as all clinical parameters listed in Table 1. Results are shown as a correlogram that reports significant associations defined by the subjective criteria Q-value < 0.05 (*p*-value < 0.01) between significantly discriminating lipids and clinical parameters, namely (**A**) blood/iron and (**B**) heart parameters. The color of circles corresponds to the direction of the relationship, namely blue for positive and red for negative, and the color intensity reflects the strength of the correlation (Pearson’s R coefficient).

Major significant changes in circulating lipid (sub)classes for female TDT patients vs. controls can be summarized as follows: (i) higher in TDT (71 species in total): 90% (64) are glycerolipids, mainly triacylgycerols (TGs; 57 species in total), but also encompass acylcarnitines (ACs; 3) with long (AC14:1 and AC18:0) and very-long (AC26:0) chain and a free fatty acid (arachidonic acid), and (ii) lower in TDT (63 total): 54% (34) are diacylglycerophospholipids, of which 21 are of the ether form, namely ether diacylglycerophosphatidylcholine and diacylglycerophosphatidylethanolamines (PCO- and PEO-, respectively), 37% (23) are sphingolipids, predominantly sphingomyelins (SMs; 22), and the remaining are cholesteryl esters (5). Of note, none of the acylcarnitines showed a significant association with any of the blood parameters measured using the selected criteria (Figure 2a). Collectively, the observed changes in circulating lipid (sub)classes in thalassemia patients recapitulate a pattern reminiscent of that reported for patients with cardiometabolic or mitochondrial diseases and are reflecting lipid metabolic dysfunction in both mitochondria (e.g., for AC14:1 and AC18:0) and peroxisomes (e.g., for AC26:0 and ether diacylglycerophospholipids, which are plasmalogens) [24,25,26,27,28].

### 2.3. Circulating Lipids Discriminating Female TDT Patients from Controls Are Differentially Correlated to Clinical Parameters

In order to determine if changes detected in the circulating lipidome in TDT patients correlated with clinical data parameters, a correlation matrix was constructed using the 136 annotated lipids that are significantly different between female TDT patients and controls with a Q-value < 0.01 reported and all parameters listed in Table 1. Figure 2 shows correlograms for significant associations based on the subjective criteria Q-value < 0.05 (equivalent to *p*-value < 0.01) between lipids and blood/iron (Figure 2a) and heart functional parameters (Figure 2b). While numerous significant associations were found, only one lipid, a cholesterol ester (CE22:6), was associated with the iron status (R = −0.49: red circles). Lipids from other (sub)classes showed a general pattern in which significant correlations were observed with different groups of clinical parameters, for example blood parameters for ether PC or PE (predominantly positive; blue circles, R from 0.65 to 0.34) and heart parameters for TGs (negative; red circles, R from −0.46 to −0.52).

A correlation matrix was built using the 136 annotated plasma lipids that discriminated female thalassemia patients from controls with a Q-value < 0.01 and FC > 2 or <0.5, as well as all clinical parameters listed in Table 1. Results are shown as a correlogram that reports significant associations defined by the subjective criteria Q-value < 0.05 (*p*-value < 0.01) between significantly discriminating lipids and clinical parameters, namely (A) blood/iron and (B) heart parameters. The color of circles corresponds to the direction of the relationship, namely blue for positive and red for negative, and the color intensity reflects the strength of the correlation (Pearson’s R coefficient).

### 2.4. Two Circulating Protein Biomarkers Related to Cardiometabolic Disease Are Elevated in Female TDT Patients Versus Controls and Correlated with Blood Parameters and Circulating Ether PCs

**Figure 3:**
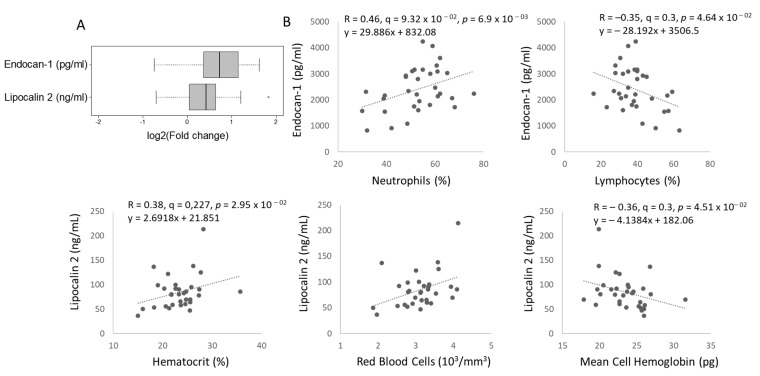
Serum biomarkers Lcn2 and endocan-1 are elevated in female thalassemia patients and differentially associated with blood parameters. (**A**) Box plots of serum Lcn2 and endocan-1 in female thalassemia patients (n = 33) versus healthy female controls (n = 9) measured by enzyme-linked immunosorbent assay (ELISA). (**B**) Pearson correlations between Lcn2 or endocan-1 and selected blood parameters.

**Figure 4:**
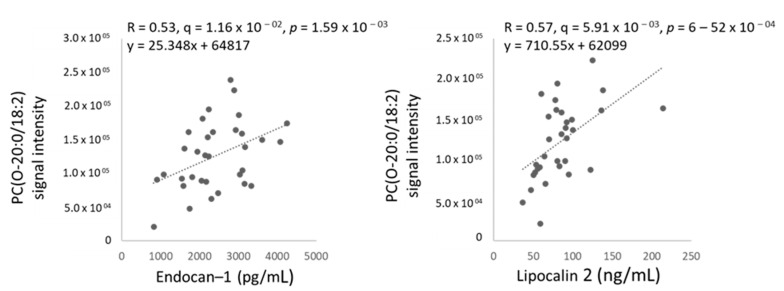
Serum biomarkers Lcn2 and endocan-1 are associated with ether diacylglycerophosphocholine (PCO-) in female thalassemia patients. Pearson correlations between Lcn2 and endocan-1 and a selected ether diacylglycerophosphocholine PC(O-20:0/18:2).

Next, plasma samples from female TDT patients and healthy female controls were characterized for 15 serum biomarkers known to be associated with cardiometabolic disease (Supplementary Table S1), from which only two were significantly elevated in TDT patients, namely endocan-1 and Lcn2, shown using box plots as FC versus controls in Figure 3a. Further to this finding, we tested for associations between serum biomarkers and (i) clinical parameters and (ii) lipids significantly discriminating female thalassemia patients vs. controls (with Q-value < 0.01 in Figure 1). Supplementary Figures S2 and S3 report results for associations (positive in blue, negative in red) between all measured serum biomarkers and clinical parameters (Q-value < 0.3, corresponding to *p*-value < 0.05) or lipids significantly discriminating thalassemia patients from controls (Q-value < 0.05, corresponding to *p*-value < 0.01). Figure 3b and Figure 4 show significant linear associations between the two serum biomarkers that significantly differed between female TDT patients and controls, namely endocan-1 or Lcn2, and blood parameters (Figure 3b) or lipids (Figure 4), respectively. Interestingly, endocan-1 was associated with white blood cells, namely neutrophils (positive) and lymphocytes (negative), while Lcn2 was specifically associated with blood parameters related to red cells, namely mean hematocrit and red blood cells (positive), as well as mean cell hemoglobin (negative) (Figure 3b). Finally, Lcn2 or endocan-1 were found to be strongly associated with 3 species of the lipid (sub)class ether PCs (Supplementary Figure S3). Of note, one of them, PC(O-20:0/18:2), was strongly correlated with both endocan-1 and Lcn2 (Figure 4), while the other two, PC(O-22:1/22:6) and PC(O-24:1/22:6), were only associated with Lcn2 (Supplementary Figure S3). Data on other biomarkers tested are shown in Supplementary Table S1. For example, we hypothesized initially that adiponectin may be a useful biomarker, but no difference in total adiponectin levels was seen between control and TDT patients (Supplementary Table S1), although a significant correlation was seen between adiponectin level and LV diastolic volume (Supplementary Figure S2).

## 3. Discussion

β-thalassemia is a monogenic heritable blood disorder with significant variability in disease progression and success of treatment [29,30,31]. Thus, development of novel therapeutics, ideally matched with precision medicine based upon new biomarkers, is desirable. Untargeted lipidomics approaches have emerged as a useful tool for the discovery of underlying disease mechanisms and novel biomarkers [32,33]. Only a few metabolomics studies have been conducted in thalassemia patients, yet none used a comprehensive untargeted lipidomic approach, and only one study was conducted using adult thalassemia patients [17,34,35,36].

Through the application of a validated comprehensive untargeted lipidomic workflow using plasma samples from male and female TDT patients as well as female healthy controls, we identified major perturbations in the circulating lipidome of female TDT patients compared to healthy female controls. Lipid (sub)classes that were predominantly altered are TGs (higher), the plasmalogen precursors, namely ether PCs or PEs (PCO- and PEO-) species (lower), and sphingomyelins (lower), but included also acylcarnitines and a free fatty acid (higher), as well as PC and cholesteryl esters (lower). Of note, we did not find any significant difference in the lipidomic signature of male and female TDT patients, at least not using the selected threshold (Q-value < 0.05), suggesting that TDT mitigates at least to some extent the previously reported effect of biological sex on the plasma lipidome in healthy subjects using the same lipidomic workflow [23].

Our findings, and previous studies, regarding TGs, sphingomyelins, and acylcarnitines, convey the important metabolic perturbations that occur in thalassemia patients [17,36,37,38,39] and also extend our knowledge about their relationship to clinical parameters. Glycerolipids are the lipid class that were most affected in thalassemia patients, representing about 50% of all lipid species of the observed lipidomic signature, of which 90% were TGs. In a study of thalassemia children before and after hydroxyurea treatment, it was found that before treatment, circulating glycerolipids were significantly elevated, most likely due to the need to compensate for fatty acid synthesis [17]. In this study, all circulating TG species were elevated in TDT patients: they were of various chain length, ranging from 45 to 60 carbons, and degree of unsaturation, from 0 to 7 (Supplementary Excel File). Interestingly, correlations between TG species and clinical parameters differed according to their number of un-saturations: those associated with blood parameters, specifically platelets (negative), had generally more carbons and un-saturations, while those associated with heart functional parameters (negative) had a lower number of carbons and un-saturations (0–2). Interestingly, many TG species that are part of the TDT signature were identified as being predictive of increased diabetes risk by Rhee et al. [40] and linked to insulin resistance, namely those with 44-52 carbons and 0–2 double bonds. In contrast, we did not find any TGs with 56–60 carbons and 9–12 double bonds, which were identified as predictive of decreased diabetes risk [40].

Sphingolipids represented 18% of all lipids of the signature, of which all but one were spingomyelins (23 species in total). Sphingomyelins are a plasma membrane component and function in many signaling pathways [41,42]. Our findings of lower circulating SMs in TDT patients are consistent with previous work [36,43]. Individuals with sickle cell disease also had lower circulating sphingomyelins and ceramides [44]. In this study, although ceramide species did not pass the stringent selected threshold of FC > 2 or FC < 0.5, four of them with acyl chains similar to SM were significantly lower in female TDT patients vs. controls, with Q-value < 0.01 and FC values ranging from 0.54 to 0.76, namely Cer(d18:1/24:0), Cer(d18:2/24:0), Cer(d18:1/24:1), and Cer(d18:1/23:0) (data not shown). Among the clinical parameters tested, only one sphingomyelin (SM(d18:2/18:1)) was found to be significantly associated with two blood and heart functional parameters.

Regarding other lipid sub(classes), plasmalogens or ether glycerophospholipids (alkyl/plasmanyl- and alkenyl/plasmenyl-GP) represented 16% of the total, or 22 species. Ether lipids have been the subject of several recent reviews [37,45], potentially relevant in thalassemia due to (i) antioxidant properties due to the presence of the vinyl ether bond in alkenyls, (ii) polyunsaturated fatty acid (PUFA) source, and (iii) immune cell viability. Numerous studies have reported low circulating ether phospholipids or plasmalogens in patients with diseases associated with mitochondrial or peroxisome dysfunction [24,38,46] or leukocyte mitochondrial damage [39]. A lower level of circulating plasmalogens, namely ether PC or PEs, has not been previously reported in thalassemia patients. Of note, commonly studied and reported ether lipids have acyl chains in the *sn1* position with 16 or 18 carbons, while those in *sn2* position are polyunsaturated fatty acids. However, we found ether PCs and PEs containing acyl chains in *sn1* with 20 and 24 carbons, which were saturated or mono-unsaturated, for which much less is known about their biological role. These species were reported in two other patient populations, namely patients with non-alcoholic fatty liver disease [47] and with pancreatic cancer [48]. Their lower level in thalassemia patients could therefore be linked to pathological mechanisms, namely high oxidative stress or inflammatory status [49,50], and/or reflect a prevailing mitochondrial/peroxisomal dysfunction in one or more organs. An intriguing emerging theme of research is the significance of ferroptosis in cardiometabolic diseases, particularly relevant to this study since a recent paper found that low levels of ether lipids PUFAs may protect against cell susceptibility to ferroptosis [51]. Of particular interest in our current study, most of the circulating ether PC or PE were significantly and differentially associated with specific clinical parameters, both blood and heart parameters, emphasizing their relevance to the status of TDT patients.

Intriguingly, the global pattern of altered lipidomic signature in patients with TDT is highly reminiscent of what has been reported for patients and models of many common metabolic diseases [25,26,27] as well as in a monogenic mitochondrial disorder [24]. Our data suggests that in TDT patients, lipid metabolic dysfunction occurs in mitochondria, as indicated by the elevation of long-chain acylcarnitines (AC14:1 and AC18:0), which are recognized proxies of fatty acid oxidation defects. Also in peroxisomes, as indicated by the lowering of the plasmalogen precursors, are ether PCs and PEs, but also an elevation of acylcarnitine C26:0 [24,28]. The latter organelle is increasingly recognized for its crucial role in several biological functions, including iron metabolism [52]. It is noteworthy that only one lipid species, namely the cholesterol ester (CE22:6), was significantly lower in thalassemia patients. This was found to be significantly negatively correlated with ferritin level.

Given that the TDT lipid signature was reminiscent of patients with cardiometabolic diseases, we also assessed 15 serum biomarkers recognized for their role in these diseases in female TDT patients and healthy control patients. Of the biomarkers tested, both Lcn2 and endocan-1 were significantly increased. Lcn2 (also often termed neutrophil gelatinase-associated lipocalin (NGAL)) is abundantly produced from neutrophils and adipocytes. Recent studies show that Lcn2 is a proinflammatory marker positively associated with insulin resistance and obesity-related metabolic disorders [53,54,55,56,57,58,59,60,61]. Measurement of serum Lcn2 is currently a principal diagnostic test in kidney failure [62] and recently, use of Lcn2 as a biomarker for evaluating obesity-related cardiovascular diseases has also been proposed. Lcn2 levels are also strongly associated with heart failure [53,55,63,64,65]. For example, Lcn2 is significantly augmented in patients with coronary heart disease and myocardial infarction [56,66]. Our findings are consistent with previous studies in which patients with β-thalassemia were found to have increased levels of circulating Lcn2 [13,67]. We found association of Lcn2 with several blood parameters, yet no statistically significant correlation with cardiac function in the TDT patients studied here, and this is in keeping with altered Lcn2 levels leading to cardiac remodeling which precedes overt functional changes. Our data is in agreement with one previous study which observed higher Lcn2 levels in b-thalassemia major patients versus healthy controls, and that it proven to be a biomarker of inflammation and early renal injury [68]. Previous studies have identified endocan-1 as a potential biomarker for cardiovascular diseases [69,70,71]. Endocan-1 is released by vascular endothelial cells, and increased levels are an indicator of endothelial dysfunction [70]. Our study is the first to identify that endocan-1 is significantly elevated in thalassemia patients, and the relationship with clinical characteristics and outcomes must now be further investigated. Interestingly, both Lcn2 and endocan-1 showed a significant positive correlation with four ether PCs, thereby linking them to specific lipid perturbations in the peroxisome. Of note, all these ether PCs have unusual fatty acyl chains in *sn1* position with 20 carbons or more. A previous study found that injection of sodium valproate (SVP) into rats led to an increase in hepatic iron, which was reversed by co-injection of the ginsenoside compound K. Proteonomic analysis revealed that the protective effects were in part due to change in the peroxisome pathway as well as changes in hepcidin [72]. Recently, it was found that altered level of transferrin receptor 1 (TfR) impacted peroxisome function and increased resistance to oxidative stress [73]. Taken together, these studies highlight the possible important role of peroxisomes in regulating whole-body iron metabolism.

In conclusion, we identified a previously uncharacterized circulating lipidomic signature in TDT characterized by changes in TGs (elevated), sphingomyelins and plasmalogens, ether PCs and PEs (reduced), but also PCs (mostly reduced), acylcarnitines (elevated), and cholesterol esters (reduced). These were differentially associated with clinical parameters, namely those reflecting blood, iron, and cardiac status. Male and female TDT patients displayed a similar lipid signature. Collectively, the lipidomic signature is reminiscent of that of patients with cardiometabolic diseases and reflecting lipid metabolic dysfunction in both mitochondria and peroxisomes. Additionally, two biomarkers (Lcn-2 and endocan-1) were identified as relevant biomarkers for the thalassemia patient population, which correlated differentially with specific blood parameters, and with a lipid (sub)class, namely ether PCs. Overall, this study provides new insight on plasma lipid and endocrine changes in β-thalassemia patients and we propose that these may have significant potential in both predictive diagnostics and therapeutics.

## 4. Materials and Methods

### 4.1. Human Cohort of TDT Patients and Control Healthy Subjects

The study protocol was approved by the Institutional Ethics Committee of the Faculty of Medicine, Chiang Mai University, Chiang Mai, Thailand. The inclusion criteria included: being diagnosed with TDT, having regular red blood cell transfusions at least once a month after diagnosis, being in the age range from 18 to 50 years, and having no infectious diseases or acute illnesses at beginning of the investigation. The exclusion criteria included patients with a contraindication to magnetic resonance imaging (MRI), and clinical evidence of other secondary causes of pulmonary hypertension, including human immunodeficiency virus (HIV) infection, hepatitis virus infection, collagen vascular diseases, cirrhosis, chronic obstructive airway diseases, and acquired heart disease associated with pulmonary hypertension. Patients with neurological diseases causing dysautonomia, severe depression, or chronic inflammatory diseases, as well as patients who had known cases of the diseases that can be the causes of diastolic and systolic cardiomyopathy (e.g., diabetes, hypertension, or coronary heart disease) were also excluded. Additionally, 10 control healthy subjects (9 female and 1 male) were recruited, and serum was also collected from these individuals. The body weight, height, and body mass index (BMI) were measured in each patient as described previously [74]. Routine laboratory tests, serum ferritin, plasma NTBI, echocardiography, 24 h Holter electrocardiogram (ECG) recording for heart rate variability (HRV) analysis, and CMR T2 * were investigated and analyzed at the time of entry. Serum ferritin and plasma NTBI were determined using standard techniques as described previously [74].

### 4.2. Sample Processing and Untargeted Lipidomic Analysis by LC-MS

Untargeted lipidomic analyses were performed using a previously validated workflow [23]. Briefly, lipids were extracted from serum after spiking with the following internal standards: LPC 13:0, PC19:0/19:0, PC14:0/14:0, PS12:0/12:0, PG15:0/15:0, and PE17:0/17:0 (Avanti Polar Lipids Inc). Sample (1 μL) was injected into a 1290 Infinity HPLC coupled to a 6530 accurate mass QTOF MS system (Agilent, Technologies Inc., Santa Clara, CA, USA) equipped with a dual electrospray ionization (ESI) source and analyzed in positive scan mode. A Zorbax Eclipse plus column (C18, 2.1 × 100 mm, particle size 1.8 μm, Agilent Technologies Inc.) was used for lipid elution over 83 min at 40 °C using a gradient of solvent A (0.2% formic acid and 10 mM ammonium formate in water) and B (0.2% formic acid and 5 mM ammonium formate in methanol/acetonitrile/methyl *tert*-butyl ether (MTBE), 55:35:10 (*v/v/v*)). MS data processing was achieved using the Mass Hunter Qualitative Analysis software package (version B.06) and a bioinformatic pipeline that we have developed which is encoded in both Perl and R languages to ensure optimal MS data alignment between chromatographic runs and perform several steps such as filter of presence, data normalization using cyclic loss, imputation of missing values using k-nearest neighbor (k = 5), and correction of batch effect with Combat. This yields a dataset listing features with their mass, corrected signal intensity, and retention time. Lipid annotation, including FA side chains, was achieved by alignment with our in-house database containing 498 lipids, which have been previously identified by tandem MS as well as by additional tandem MS, for which spectra were manually interpreted in silico, similar to Godzien et al. [75], as previously described [23]. For some ether lipids, it was difficult to distinguish plasmanyl (O-alkyl) from plasmenyl (P-alkyl) glycerophospholipids or plasmalogens since MS/MS identifies the number of un-saturations on acyl chains, but not their position. Hence, ether lipids were annotated as “O-alkyl” and referred to as ether phospholipids.

### 4.3. Biomarker and NTBI Analyses

We used enzyme-linked immunosorbent assay (ELISA) kits to measure Lcn2 and adiponectin (ImmunoDiagnostics Ltd) as well as IL-1beta (Thermofisher Scientific) following manufacturer’s instructions. Multiplexing MILLPLEX Human Cardio Array 12-plex (Millipore) using the Luminex™ 100 system analysis of cytokines, chemokines, and growth factors was performed by Eve Technologies Corp. (Calgary, AB, Canada) using a Bio-PlexTM 200 system (Bio-Rad Laboratories Inc., Hercules, CA, USA). In total, 12 cardiac biomarkers were measured (brain natriuretic peptide (BNP), N-terminal pro b-type natriuretic peptide (NTproBNP), Creatine kinase-MB (CK-MB), C-X-C Motif Chemokine Ligand 6 (CXCL6), C-X-C Motif Chemokine Ligand 16 (CXCL16), Endocan-1, Fatty-acid-binding protein 3 (FABP3), Fatty-acid-binding protein 4 (FABP4), Phosphatidylinositol-glycan biosynthesis class F (PIGF), tumor necrosis factor superfamily member 14 (LIGHT), Oncostatin M (OSM), Troponin I). Results were expressed as pg/mL of plasma. To determine NTBI, blood was collected using a heparin-coated syringe followed by use in the method of nitrilotriacetic acid (NTA) disodium salt chelation/flow cytometry. First, Fe^3+^-(NTA)_2_ complex was generated by incubating plasma with NTA solution (80 mM) at 24 °C for 30 min followed by separation of the complex from plasma by centrifugation through a 30 kDa cut-off membrane filter (NanoSep^®^, Pall Life Sciences, Ann Arbor, MI, USA). The concentrations of Fe^3+^-(NTA)_2_ in the ultrafiltrate, which represent the plasma NTBI levels, were then determined via flow cytometry using chelatable fluorescent beads (Guava EasyCyte HT, Merck Millipore, Germany). A standard curve was produced and used for accurate determination of the plasma NTBI level [76].

### 4.4. Data Analysis and Statistics

Data for clinical parameters and serum biomarkers are reported either as mean ± standard deviation (SD), or in the case of data not passing the D’Agostino and Pearson omnibus normality test, data were reported and median (min, max). Significance for biomarker data was conducted using T-tests, as indicated. For lipidomics, independent testing of each feature was achieved using regression analysis corrected for age in R, with Storey correction for multiple comparisons using the Q-value package from Bioconductor. Data were also submitted to unsupervised principal component analysis (PCA) as well as Pearson correlation analyses. To test for correlations between lipids, biomarkers, and clinical parameters, a correlation matrix was created in R. We report results after application of a selected threshold Q-value, as an estimation of false discovery rate (FDR), as indicated, and for which we also report *p*-values. Data were adjusted for age and are depicted as volcano plots and box plots, where the midline represents the median fold change (FC) vs. controls, the box represents the interquartile range (IQR) between the first and third quartile, and whiskers represent the lowest or highest values.

## Figures and Tables

**Table 1:** Demographic, clinical, and biochemical characteristics of male and female transfusion-dependent thalassemia (TDT) patients. Characteristics of the study population of male (n = 28) and female (n = 33) thalassemia patients. Data is expressed as either mean ± standard deviation (SD) or median (min, max). Significant *p*-values (*p* < 0.05) are indicated in bold.

	Male		Female		*p*–Value
	Mean/Median	Deviation	Mean/Median	Deviation	
Number	28		33		
Age (years)	27	(18–45)	27	(19–49)	0.6155
Body weight (kg)	49	7	46	7	0.1960
Height (cm)	160	8	154	7	**0.0012**
*BMI*	19	2	20	2	0.1667
	**Blood Parameters**				
Hemoglobin (g/dL)	7.0	1.1	7.1	1.0	0.5875
Hematocrit (%)	23	4	23	4	0.9719
Platelets (10^6^/mm^3^)	527	255	435	276	0.1951
WBC (10^6^/mm^3^)	14	(6–33)	12	(3–30)	0.1459
Neutrophils (%)	46	12	53	11	**0.0184**
Eosinophils (%)	3.4	(0.7–50)	1.5	(0–8)	**0.0392**
Basophils (%)	1.7	(0.2–8.4)	0.60	(0–4)	**0.0119**
Lymphocytes (%)	38	16	39	11	0.8875
Monocytes (%)	7.6	3.9	5.6	2.1	**0.0203**
Red Blood Cells (10^3^/mm^3^)	3.1	0.7	3.1	0.5	0.8987
Mean Corpuscular Volume (μm^3^)	79	(57–91)	74	(27–87)	0.3822
Mean Cell Hemoglobin (pg)	23	3	23	3	0.4947
Mean Corpuscular Hemoglobin Concentration (g/dL)	30	(25–34)	31	(18–34)	0.5327
Red blood Cell Distribution Width (%)	25	4	25	6	0.6800
Mean Platelet Volume (μm^3^)	8.1	1.4	8.2	1.4	0.8374
	**Iron Status**				
NTBI (μM)	6.5	2.31	7.2	1.7	0.2277
Ferritin (ng/dL)	1375	(434–6079)	1499	(274–7371)	0.8686
CMR T2* (ms)	37	10	40	15	0.4430
	**Heart Function**				
Heart rate (BPM)	91	14	96	12	0.1851
Systolic BP (mmHg)	111	13	109	11	0.4455
Diastolic BP (mmHg)	60	(43–99)	64	(54–88)	0.1572
LV diastolic volume (ml)	102	21	79	17	**<0.0001**
LA size (mm)	3.8	0.39	3.4	0.45	**0.0008**
LV EF (%)	69	4.4	69	3.5	0.4861
TRV max	259	(191–452)	236	(189–536)	0.3482
E/A ratio	1.6	(0.9–2.8)	1.7	(0.8–3.5)	0.6379
S (tissue doppler)	9.2	(6.9–11.7)	8.9	(5.2–11.1)	**0.0475**
E (tissue doppler)	11	2.0	11	2.6	0.3499
A (tissue doppler)	8.3	2.1	7.7	2.0	0.2789
SDNN (ms)	100	27	94	29	0.4206
SDANN (ms)	92	27	88	30	0.6033
ASDNN (ms)	39	11	33	11	0.0628
rMSSD (ms)	21	(9–46)	17	(9–43)	0.1506
LF (ms^2^)	13	(5–21)	10.0	(4–24)	0.0808
HF (ms^2^)	8.5	(2.7–18.6)	6.3	(2.1–17.2)	0.2540
LF/HF ratio	1.6	0.46	1.5	0.32	0.2295

BMI, body mass index; WBC, White Blood Cells; NTBI, Non Transferrin Bound Iron; CMR T2*, Cardiovascular Magnetic Resonance T2*; BPM, Beats Per Minute; BP, Blood Pressure; LV, Left Ventricular; LA, Left Atrial; EF, Ejection Fraction; TRV max, Tricuspid Regurgitation Velocity max; S, Systolic; SDNN, Standard deviation of the R-R intervals over a 24-h period; SDANN, standard deviation of all 5-min mean R-R intervals; ASDNN, average standard deviation of all 5-min R-R intervals; rMSSD, square root of mean squared differences of successive R-R intervals; LF, Low Frequency; HF, High Frequency.

## Data Availability

All clinical data are provided in the results of the manuscript and full lipidomic analysis datasets are provided in two supplementary excel files.

## Supplementary Materials

The following are available online at https://www.mdpi.com/2218-1989/11/2/70/s1, Figure S1: Volcano plot for lipidomic analysis in male vs female patients, Figure S2: Serum biomarkers and clinical (blood/iron and heart) parameters, Figure S3: Serum biomarkers and circulating discriminating lipids, Table S1: clinical biomarkers of TDT and control patients, Supplementary Excel file Sheet 1: List of the 387 features significantly (*p* < 0.03, FC > 2 and Q-value < 0.01) different between female TDT patients vs. healthy controls of which 136 were annotated using a combination of our in-house reference database and tandem MS analysis, Supplementary Excel file Sheet 2: List of 105 features that passed the criteria (*p* < 0.05, FC > 2 and Q-value < 0.53) for the comparison between female vs. male TDT patients of which 21 were annotated using a combination of our in-house
