# Supplementary material for: Identification of Circulating Endocan-1 and Ether Phospholipids as Biomarkers for Complications in Thalassemia Patients
Source: Metabolites. 2021 Jan 26;11(2):70. doi: 10.3390/metabo11020070 (PMC7912378; doi:10.3390/metabo11020070)
Supplement: Supplementary file 1 [file metabolites-11-00070-s001.zip › metabolites-1029505-supplementary.docx]

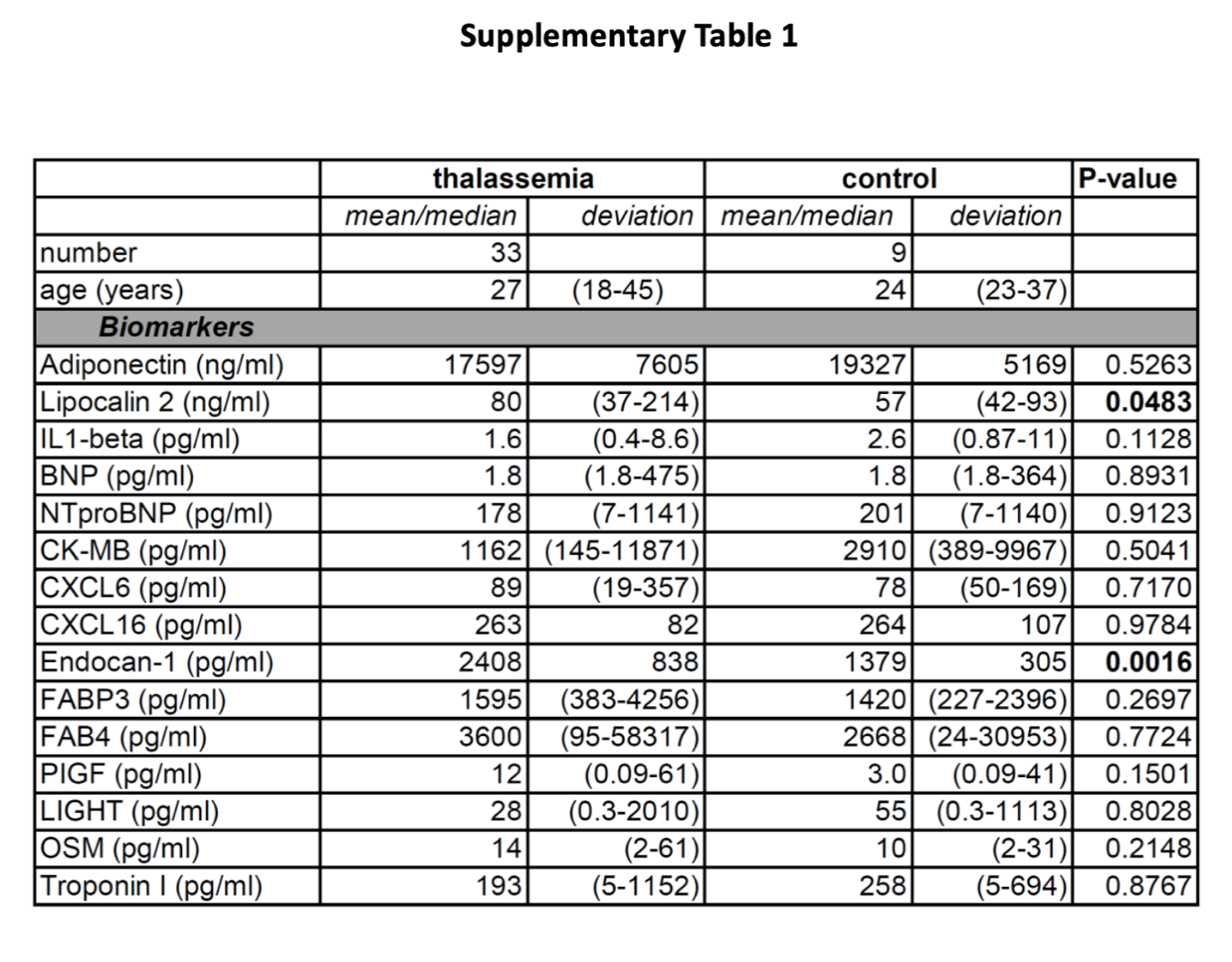


**Suppl. Table 1. Comparison of serum biomarkers in female TDTM patients and healthy female controls.**

Serum biomarkers from female TDTM (n=33) and healthy female controls (n=9). Significant P-values (p<0.05) are indicated in bold.


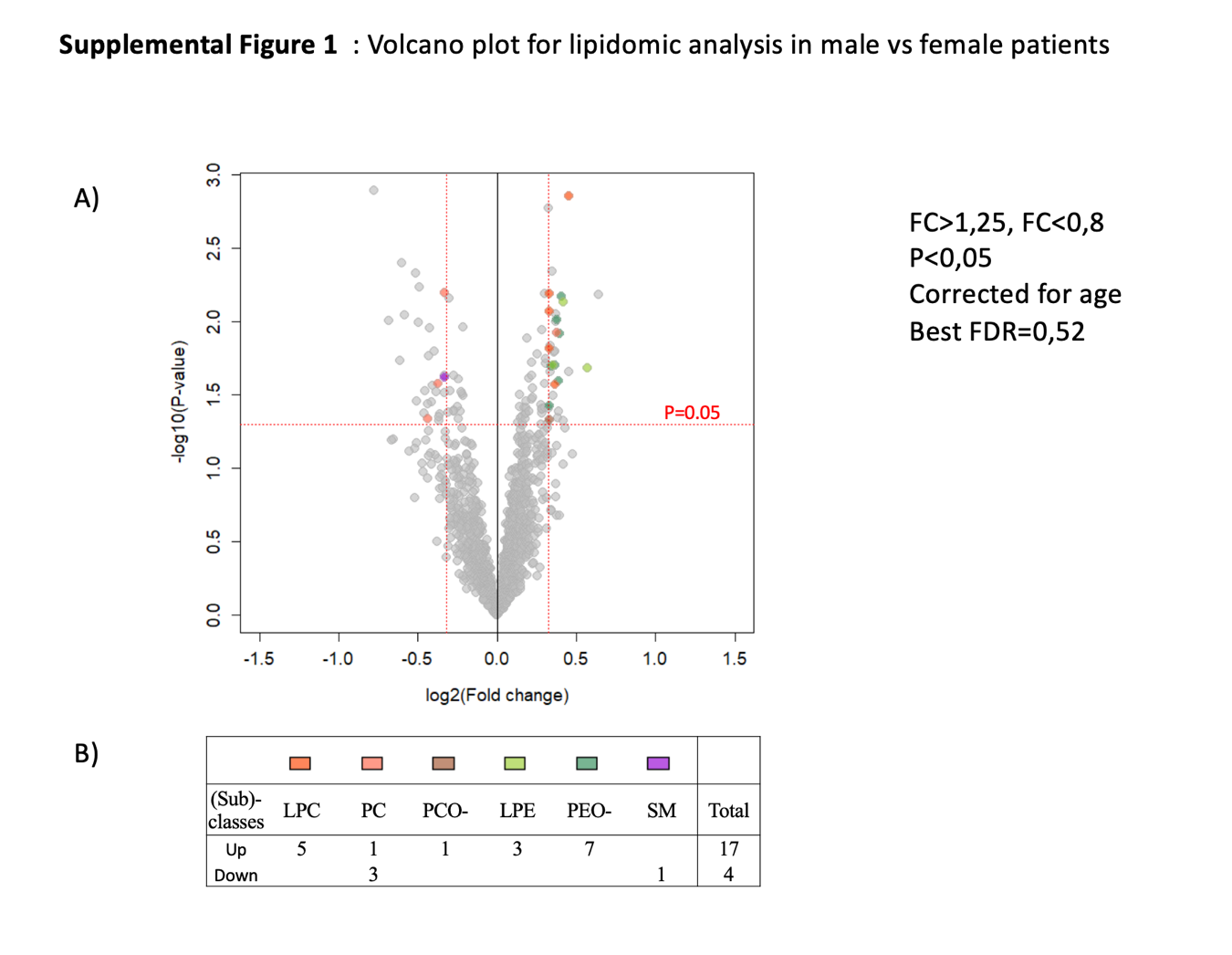


**Suppl Fig 1.** **Untargeted lipidomic analysis of serum from male and female TDTM patients showed no significant differences in the lipid profile.**

Volcano plot from LC-QCTOF based untargeted lipidomics of plasma from male (n=28) and female (n=33) TDTM patients, depicting the 1,463 features obtained following MS data processing. The x axis represents fold changes (FC; log2) of MS signal intensity values in male vs. female patients, with the y axis representing *P* values (-log10). There were 59 features that passed the selected threshold of P-value < 0.05 (corresponding to Q-value<0.53; horizontal red line; corQv alues) and FC>1.25 or FC<0.8 (vertical red lines), of which 21 unique lipids were annotated using our in-house reference database as shown by the color symbols. All 59 features had Q-values between 0.52 and 0.53.


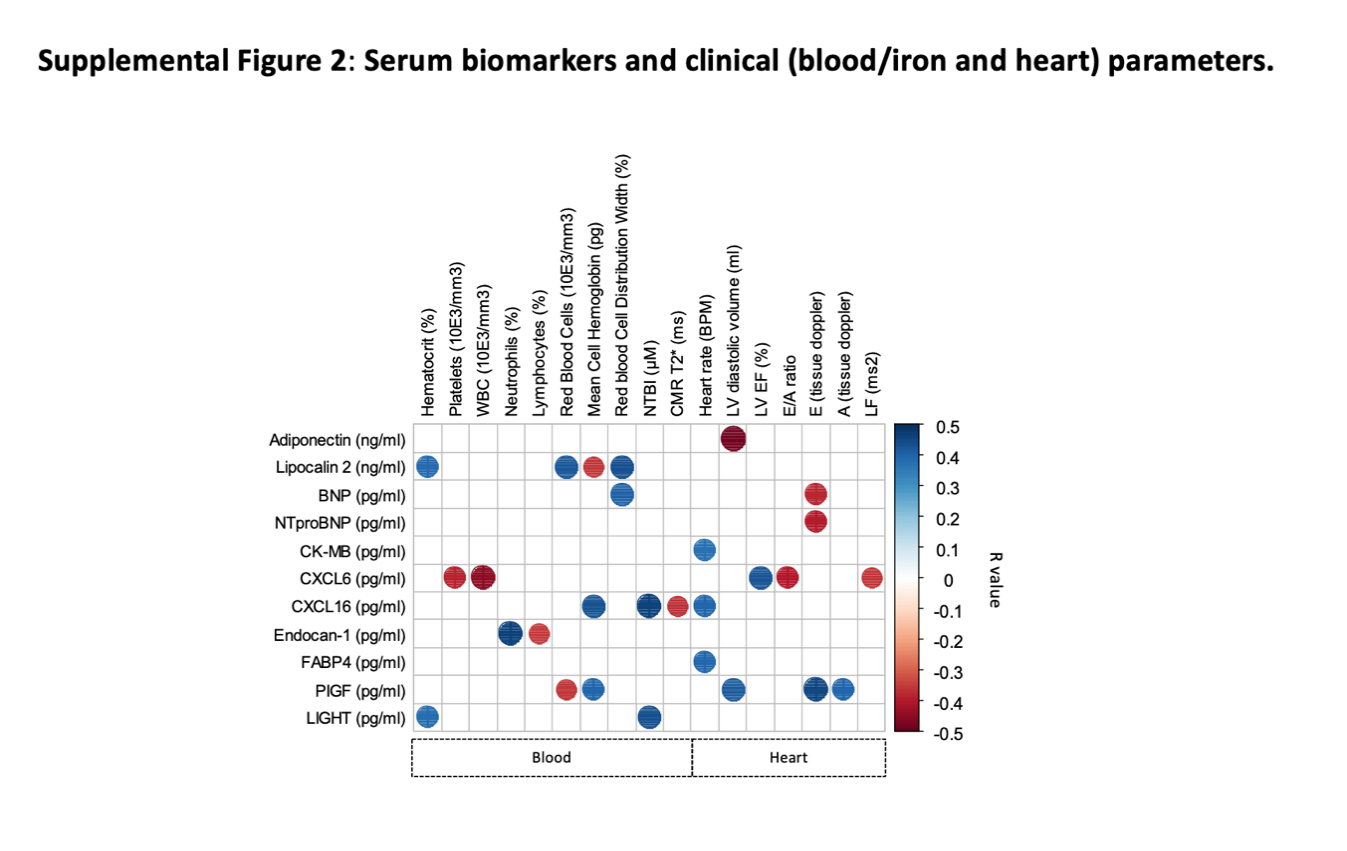


**Supp Figure 2. Serum biomarkers are differentially correlated to clinical parameters.**

A correlation matrix was built using the all measured serum biomarkers listed in Suppl. Table 1 and the all clinical parameters listed in Table 1. Results are shown as correlogram that reports significant correlations defined by the subjective criteria Q-value<0.3; corresponding to P-value<0.05 between biomarkers and clinical parameters. The colour of circles corresponds to the direction of the relationship, namely blue for positive and red for negative, and the colour intensity reflects the strength of the correlation (Pearson R coefficient).


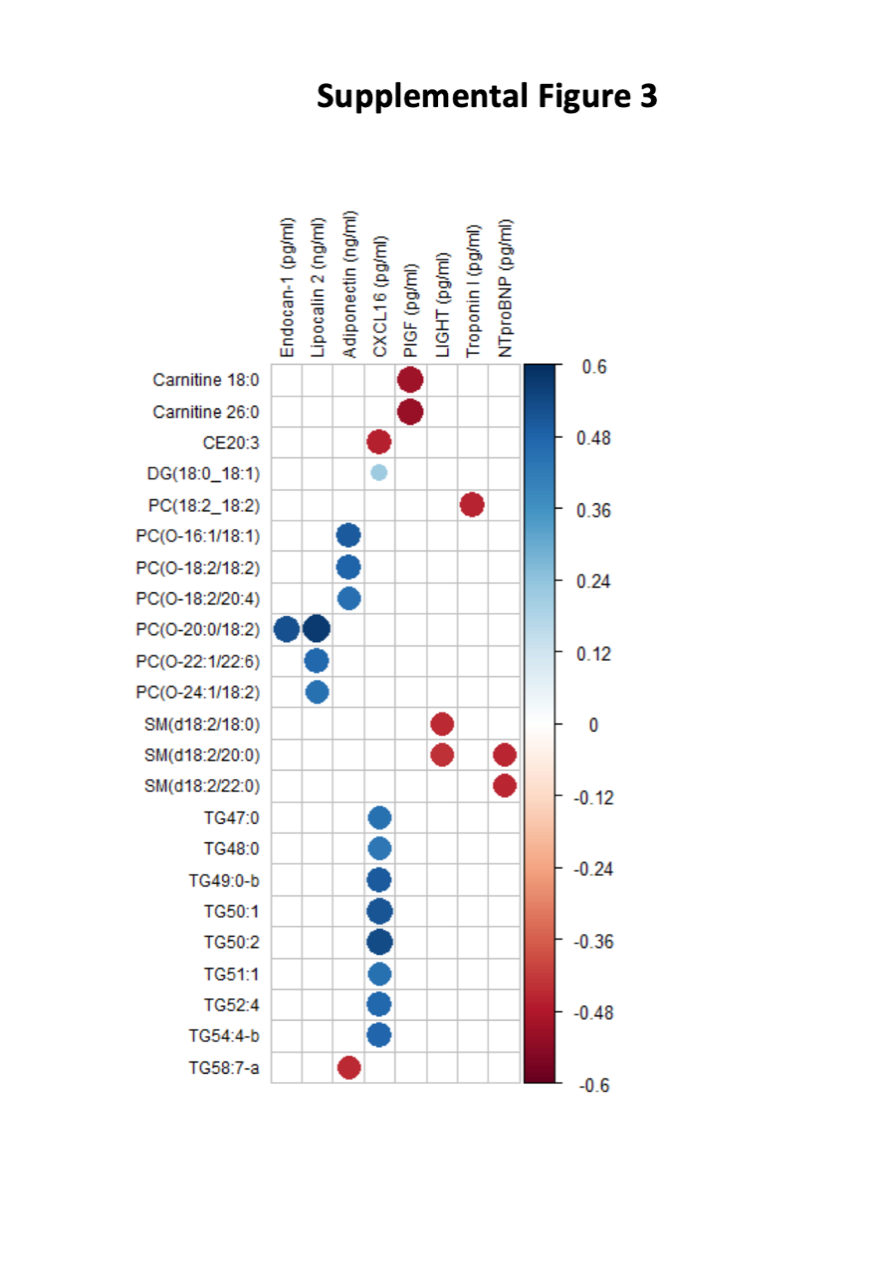


**Supp Figure 3. Lipids are differentially correlated to serum biomarkers.**

A correlation matrix was built using the 136 annotated plasma lipids that discriminated female thalassemia patients from controls with a Q-value < 0.01 and FC>2 or <0.5 as well as all biomarkers listed in Suppl Table 1. Results are shown as correlogram that reports significant correlations defined by the subjective criteria Q-value < 0.05 (P-value < 0.01) between significantly discriminating lipids and biomarkers. The colour of circles corresponds to the direction of the relationship, namely blue for positive and red for negative, and the colour intensity reflects the strength of the correlation (Pearson R coefficient).

**Suppl Excel File 1:**

List of the 387 features significantly (P<0.03, Q-value <0.01, and FC>2 or FC<0.5) different between female TDTM patients vs. female healthy controls of which 136 were annotated using a combination of our in-house reference database and tandem MS analysis. The table list for each features the following values: P-values, Q-values and fold-change (FC) vs. controls, as well as the mean and SD for their signal intensity.

**Suppl Excel File 2:**

List of 59 features that passed the criteria (P<0.05, Q-value <0.53, FC>1.25 or FC<0.8) for the comparison between female vs. male TDTM patients of which 21 were annotated using a combination of our in-house reference database and tandem MS analysis. The table list for each features the following values: P-values, Q-values and fold-change (FC) vs. male, as well as the mean and SD for their signal intensity.
